# Genotypic characteristics of *Mycobacterium* tuberculosis isolated from household contacts of tuberculosis patients in the Philippines

**DOI:** 10.1186/1471-2334-13-571

**Published:** 2013-12-05

**Authors:** Irene G Sia, Seanne P Buckwalter, Kelly A Doerr, Sonia Lugos, Rebecca Kramer, Ruth Orillaza-Chi, Maria Imelda Quelapio, Thelma E Tupasi, Nancy L Wengenack

**Affiliations:** 1 Department of Laboratory Medicine and Pathology, Mayo Clinic, 200 First Street SW, Rochester, MN 55905, USA; 2 Michigan Department of Community Health, Lansing, Michigan, USA; 3 University of the Philippines and Philippine General Hospital, Manila, The Philippines; 4 Tropical Disease Foundation, Manila, The Philippines; 5 Makati Medical Center, Manila, The Philippines; 6 Division of Infectious Diseases, Mayo Clinic, 200 First Street SW, Rochester, MN 55905, USA

## Abstract

**Background:**

The Philippines has an extremely high rate of tuberculosis but little is known about *M. tuberculosis* genotypes and transmission dynamics in this country. The aim of this study was to determine the proportion of household contacts who develop active TB due to direct transmission from an index case in that household.

**Methods:**

*Mycobacterium tuberculosis* isolates from household contacts of tuberculosis patients in the Philippines were characterized using restriction-fragment-length polymorphism analysis, spoligotyping, and mycobacterial interspersed repetitive units – variable number tandem repeats typing (12-loci) to determine their utility in elucidating transmission in an area of high tuberculosis prevalence. Drug susceptibility patterns for these isolates were also determined.

**Results:**

Spoligotyping and MIRU-VNTR typing results matched in 10 (62.5%) of 16 index patient-household contact pairs while IS6110 fingerprints matched in only six (37.5%) pairs. Only 3/16 (18.8%) index patient-household contact pairs had identical drug susceptibility results.

**Conclusions:**

Strain typing of *M. tuberculosis* isolates from household contacts in the Philippines indicates that transmission of strains does not necessarily occur directly from the index patient living in close proximity in the same household but rather that community-based transmission also frequently occurs. Accurate susceptibility testing of all isolates is necessary to insure optimal care of both the index patients and any culture-positive household contacts.

## Background

Tuberculosis (TB) is a serious global health problem, with the largest number of cases disproportionately occurring in developing countries. The Philippines is one of the countries hardest hit by the disease, with the second highest TB incidence in Asia [1]. We previously reported a remarkably high prevalence of 12.8% for TB disease and 65.6% for TB infection among household contacts of tuberculosis patients in the Philippines [2]. The Philippines also bear a significant proportion of the global multidrug resistant (MDR) TB burden [3,4]. Although the Philippines have an extremely high rate of TB, little is known about *M tuberculosis* genotype and transmission dynamics in this country. The aim of this study was to determine the proportion of household contacts who develop active TB due to direct transmission from an index case in that household.

Molecular genotyping has advanced the understanding of *M tuberculosis* transmission and is helpful in identifying transmission links in a community. Restriction fragment-length polymorphism (RFLP) analysis of the insertion sequence 6110 (IS6110) is often used for genotyping of *M tuberculosis*. However, molecular characterization using RFLP method is laborious and requires culture for several weeks to obtain large quantities of genomic DNA. In addition, RFLP has poor discriminatory power for isolates with low numbers of insertion sites [5]. The development of PCR-based typing methods has enabled rapid mycobacterial genotyping [6,7]. Spacer oligonucleotide typing (spoligotyping) is a rapid PCR-based method that examines DNA polymorphisms at the unique direct repeat (DR) region of *M tuberculosis*. It is easier to perform and requires smaller amounts of DNA than RFLP analysis but its discriminatory capacity is inferior to RFLP. Spoligotyping is useful in discriminating strains with low IS6110 copy numbers and it can be performed on nonviable organisms. Mycobacterial interspersed repetitive units (MIRU) – variable number tandem repeats (VNTR) is another PCR-based method that is easily reproducible and does not require extensive DNA purification. MIRU-VNTR [8] has become a major method for rapid, high-resolution genotyping of *M tuberculosis* complex isolates. The method relies on PCR amplification of multiple loci (12, 15 or 24 loci) using primers specific for the flanking regions of each repeat locus and on the determination of the sizes of the amplicons, which reflect the numbers of the targeted MIRU-VNTR copies [9]. Moreover, the results are expressed as numerical codes and are therefore easy to compare and exchange between laboratories [10].

Only a limited number of *M tuberculosis* isolates from the Philippines have been genotyped [11,12]; no studies have described the epidemiologic links among isolates. The aims of this study were to characterize *M tuberculosis* isolated from TB patients in Philippine households using three methods for genotyping, namely RFLP, spoligotyping, and MIRU-VNTR, and to describe the transmission and drug susceptibility patterns of these isolates.

## Methods

### Study isolates

Sputum for acid-fast bacilli (AFB) smear microscopy and culture were collected from the index case (IC) and household contact (HC) cases during a contact investigation study on sputum smear-positive TB patients seen at the Tropical Disease Foundation DOTS Clinic, Makati Medical Center, Metro Manila between 2001 and 2003 [2,13]. Two hundred eighteen index cases aged ≥18 years with sputum smear-positive TB, including 163 (74.8%) with MDR-TB, were identified. Eight hundred ninety seven of their household contacts were evaluated for TB with tuberculin skin test, chest x-ray and sputum examination. One hundred thirteen (12.8%) household contacts with TB were identified by positive chest x-rays and/or positive sputum results; of these, 20 were sputum culture-positive. A total of 29 *M tuberculosis* paired isolates were available from 13 index patients and 16 household contacts. Patients were between the ages of 10 and 60 (mean, 31) years; eight were females, 21 were males.

All sputum specimens were first processed and cultured on Lowenstein-Jensen slants and strain identification was performed in the Philippines using the nitrate and niacin tests according to established protocols [14]. The identification of the isolates was confirmed in the United States prior to genotyping studies using a nucleic acid hybridization probe specific for *M. tuberculosis* complex (GenProbe AccuProbe®, San Diego, CA). Antimicrobial susceptibility testing was performed in the Philippines and confirmed in the United States using Clinical and Laboratory Standards Institute guidelines [15] with the 1% indirect agar proportion method used for all antimicrobial agents except pyrazinamide. Pyrazinamide was tested using the VersaTREK broth system (TREK Diagnostics, Cleveland, OH). Isolates were genotyped using RFLP IS6110 fingerprinting [5], spoligotyping [6] and 12-loci MIRU-VNTR typing [16] as previously described.

### Ethics statement

The study was approved by the Institutional Review Boards of Mayo Clinic Rochester and the Tropical Disease Foundation - Makati Medical Center. Written informed consent was obtained from all patients, adult household contacts, and/or head of household.

## Results

### Genotyping

IS6110 fingerprinting, spoligotyping and 12-loci MIRU-VNTR typing were done on 29 *M tuberculosis* isolates from 13 households in the Philippines (Table 1). One household had three household contacts with TB; another household had two contacts with TB. Altogether, there were 16 paired IC-HC isolates. Genotyping found 23 IS6110 fingerprints, 9 spoligotypes, and 13 MIRU-VNTR types.

**Table 1:** **IS6110 Restriction fragment length polymorphism (RFLP), spoligotyping, and mycobacterial interspersed repetitive unit variable numbers of tandem repeat (MIRU-VNTR) typing patterns of *Mycobacterium tuberculosis *** **isolates from index case (IC) and household contact (HC) cases from the Philippines**

**Household/index group**	**RFLP Pattern**	**Spoligotype pattern**	**MIRU-VNTR Code**	**Lineage/family**	**Methods with matching paired IC-HC**	**DST match?**
		**1 Spacer # 43**				
IC1	5–13	■■□ ■■■ ■■■ ■■■ ■■■ ■■■ ■□□ ■■■ ■■■ ■□□ □□■ □■■ ■■■ ■■■ ■	244336223432	Manila		
HC1	5–13	■■□ ■■■ ■■■ ■■■ ■■■ ■■■ ■□□ ■■■ ■■■ ■□□ □□■ □■■ ■■■ ■■■ ■	244336223432	Manila	RFLP/Spoligo/MIRU	No
IC2	10–14	■■□ ■■■ ■■■ ■■■ ■■■ ■■■ ■□□ ■■■ ■■■ ■□□ □□■ □■■ ■■■ ■■■ ■	254326223432	Manila		
HC2	34–13	■■□ ■■■ ■■■ ■■■ ■■■ ■■■ ■□□ ■■■ ■■■ ■□□ □□■ □■■ ■■■ ■■■ ■	254326223432	Manila	Spoligo/MIRU	No
IC3	2–13	■■□ ■■■ ■■■ ■■■ ■■■ ■■■ ■□□ ■■■ ■■■ □□□ □□□ □□■ ■■■ ■■■ ■	254326223432	ND		
HC3	2–13	■■□ ■■■ ■■■ ■■■ ■■■ ■■■ ■□□ ■■■ ■■■ □□□ □□□ □□■ ■■■ ■■■ ■	254326223432	ND	RFLP/Spoligo/MIRU	No
IC4	35–11	■■□ ■■■ ■■■ ■■■ ■■■ ■■■ ■□□ ■■■ ■■■ ■□□ □□■ □■■ ■■■ ■■■ ■	254326223422	Manila		
HC4	3–12	■■□ ■■■ ■■■ ■■■ ■□□ □■■ ■□□ ■■■ ■■■ ■□□ □□■ □■■ ■■■ ■■■ ■	254326223432	ND	None	No
IC5	1–13	■■□ ■■■ ■■■ ■■■ ■■■ ■■■ ■□□ ■■■ ■■■ ■□□ □□■ □■■ ■■■ ■■■ ■	254326223332	Manila		
HC5	1–13	■■□ ■■■ ■■■ ■■■ ■■■ ■■■ ■□□ ■■■ ■■■ ■□□ □□■ □■■ ■■■ ■■■ ■	254326223332	Manila	RFLP/Spoligo/MIRU	Yes
IC6	11–15	■■□ ■■■ ■■■ ■■■ ■■■ ■■■ ■□□ ■■■ ■■■ ■□□ □□■ □■■ ■■■ ■■■ ■	234326223432	Manila		
HC6.1	4–16	■■□ ■■■ ■■■ ■■■ ■■■ ■■■ ■□□ ■■■ ■■■ ■□□ □□■ □■■ ■■■ ■■■ ■	234326223432	Manila	Spoligo/MIRU (IC6/HC6.1)	No
HC6.2	6–14	■■□ ■■■ ■■■ ■■■ ■■■ ■■■ ■□□ ■■■ ■■■ ■□□ □□■ □■■ ■■■ ■■■ ■	234326223432	Manila	Spoligo/MIRU (IC6/HC6.2)	No
HC6.3	7–13	□■□ ■■■ ■■■ ■■■ ■■■ ■■■ ■□□ ■■■ ■■■ ■□□ □□■ □□□ ■■■ ■■■ ■	253326223432	ND	None (IC6/HC6.3)	No
IC7	19–12	■■□ ■■■ ■□□ □□□ □□□ □□□ □□□ □□□ □□□ □□□ □□□ □□□ □□■ ■■■ ■	254326223432	ND		
HC7.1	20–13	■■□ ■■■ ■■■ ■■■ ■■■ ■■■ ■□□ ■■■ ■■■ ■□□ □□■ □■■ ■■■ ■■■ ■	254326223432	Manila	MIRU (IC7/HC7.1)	No
HC7.2	18–11	■■□ ■■■ □■■ ■■■ ■■■ ■■■ ■□□ ■■■ ■■■ ■□□ □□■ □□■ □■■ ■■■ ■	274326223432	ND	None (IC7/HC7.1)	No
IC8	12–15	■■□ ■■■ ■■■ ■■■ ■■■ ■■■ ■□□ ■■■ ■■■ ■□□ □□■ □■■ ■■■ ■■■ ■	234326223432	Manila		
HC8	37–13	■■□ ■■■ ■■■ ■■■ ■■■ ■■■ ■□□ ■■■ ■■■ ■□□ □□■ □■■ ■■■ ■■■ ■	254326223432	Manila	Spoligo	No
IC9	14–19	□□□ □□□ □□□ □□□ □□□ □□□ □□□ □□□ □□□ □□□ □□□ □■■ ■■■ ■■■ ■	223325173533	Beijing		
HC9	14–19	□□□ □□□ □□□ □□□ □□□ □□□ □□□ □□□ □□□ □□□ □□□ □■■ ■■■ ■■■ ■	223325173533	Beijing	RFLP/Spoligo/MIRU	Yes
IC10	27–12	■■□ ■■■ ■■■ ■■■ ■■■ ■■■ ■□□ ■■■ ■■■ ■□□ □□■ □■■ ■■■ ■■■ ■	254326223422	Manila		
HC10	27–12	■■□ ■■■ ■■■ ■■■ ■■■ ■■■ ■□□ ■■■ ■■■ ■□□ □□■ □■■ ■■■ ■■■ ■	254326223422	Manila	RFLP/Spoligo/MIRU	Yes
IC11	25–15	■■□ ■■■ ■■■ ■■■ ■■■ ■■■ ■□□ ■■■ □□□ □□□ □□□ □□□ □□□ □□□ □	254326224332	ND		
HC11	23–12	■■□ ■■■ ■■■ ■■■ ■■■ ■■■ ■□□ ■■■ ■■■ ■□□ □□■ □■■ ■■■ ■■■ ■	254326223432	ND	MIRU	No
IC12	32–11	■■□ ■■■ ■■■ ■■■ ■■■ ■■■ ■■□ □□□ □□□ □□□ ■■□ □□□ ■■■ ■■■ ■	224227163221	ND		
HC12	28–13	■■□ ■■■ ■■■ ■■■ ■■■ ■■■ ■■□ □□□ □□□ □□□ ■■□ □□□ ■■■ ■■■ ■	224227163321	ND	Spoligo/MIRU	No
IC13	29–11	■■□ ■■■ ■■■ ■■■ ■■■ ■■■ ■□□ ■■■ ■■■ ■□□ □□■ □■■ ■■■ ■■■ ■	354326223422	Manila		
HC13	29–11	■■□ ■■■ ■■■ ■■■ ■■■ ■■■ ■□□ ■■■ ■■■ ■□□ □□■ □■■ ■■■ ■■■ ■	354326223422	Manila	RFLP/Spoligo/MIRU	No

*RFLP* = restriction fragment length polymorphism; *Spoligo* = spligotyping; *MIRU* = multiple interspersed repeating units/variable number tandem repeats; *ND* = not determined; *IC* = index case; *HC* = household contact patient, *DST* = drug susceptibility.

When known, lineage information is listed.

Isolates had between 11 and 19 (median, 13) copies of IS6110 by RFLP. By spoligotyping, two isolates from one household (Table 1, IC9 and HC9) had the Beijing genotype and 17 (58.6%) of isolates from nine households lacked eight spacers characteristic of the Manila family [12].

Spoligotyping and MIRU-VNTR typing both matched in 10 (62.5%) of 16 pairs while IS6110 fingerprints matched in only six (37.5%) IC-HC pairs. All six pairs with matching IS6110 fingerprints also matched by spoligotypes and MIRU-VNTR types. Three paired isolates matched by only one genotyping method, and the remaining three pairs had genotypes that were different by all three fingerprinting methods.

### Drug susceptibility testing

Results of drug susceptibility testing are shown in Table 2. Only four of the 29 (13.8%) isolates were fully susceptible to all first line drugs; eight (27.6%) were resistant to all first line drugs. Isoniazid (INH) resistance was detected in 24/29 (82.8%) and 19/29 (65.5%) at low (0.2 μg/ml) and high (1.0 μg/ml) concentrations, respectively. Resistance to rifampin (RIF), ethambutol (EMB) and pyrazinamide (PZA) occurred in 19/29 (65.5%), 14/29 (48.3%), and 9/28 (32.1%), respectively. There were 19 (65.5%) MDR *M tuberculosis* isolates. With second line drugs, resistance to streptomycin (STR) at 2.0 μg/ml, streptomycin at 10 μg/ml, capreomycin (CAP), and ethionamide (ETH) was detected in 16/29 (55.2%), 13/29 (44.8%), 3/29 (10.3%), and 6/29 (20.7%), respectively. Resistance to kanamycin (KAN), amikacin (AMI), cycloserine (CYC) and para-aminosalicylic acid (PAS) was not detected. Because resistance testing to fluoroquinolones was not performed, the number of extensively-drug resistant isolates is not known.

**Table 2:** **Drug susceptibilities of *Mycobacterium tuberculosis *** **isolates from index case (IC) and household contact (HC) cases in the Philippines**

	**First line drugs**^***a***^					**Second line drugs**							
**Household/index contacts**	**INH**		**RIF**	**EMB**	**PZA**	**STR**		**CAP**	**KAN**	**AMI**	**ETH**	**CYC**	**PAS**
	**0.2**	**1.0**	**1.0**	**7.5**	**300**	**2.0**	**10**	**10**	**6.0**	**6.0**	**10**	**60.0**	**8.0**
IC1	R	R	R	R	R	S	S	S	S	S	S	S	S
HC1	R	R	S	S	S	S	S	S	S	S	S	S	S
IC2	R	R	R	R	R	R	R	S	S	S	S	S	S
HC2	R	R	R	S	S	R	R	S	S	S	S	S	S
IC3	R	S	R	S	S	R	R	S	S	S	S	S	S
HC3	R	R	R	S	S	S	S	S	S	S	S	S	S
IC4	R	R	R	S	N/A^*b*^	R	S	S	S	S	R	S	S
HC4	R	R	S	S	S	R	S	S	S	S	S	S	S
IC5	R	R	R	R	R	R	R	S	S	S	S	S	S
HC5	R	R	R	R	R	R	R	S	S	S	S	S	S
IC6	R	R	R	R	R	R	R	S	S	S	R	S	S
HC6.1	R	R	R	R	S	R	R	S	S	S	R	S	S
HC6.2	R	R	R	R	S	R	R	S	S	S	R	S	S
HC6.3	S	S	S	S	S	S	S	S	S	S	S	S	S
IC7	R	S	R	R	S	R	R	S	S	S	R	S	S
HC7.1	S	S	S	S	S	S	S	S	S	S	S	S	S
HC7.2	S	S	S	S	S	S	S	S	S	S	S	S	S
IC8	R	S	S	S	S	S	S	S	S	S	S	S	S
HC8	S	S	S	S	R	S	S	S	S	S	S	S	S
IC9	R	R	R	R	R	R	R	S	S	S	S	S	S
HC9	R	R	R	R	R	R	R	S	S	S	S	S	S
IC10	R	S	S	S	S	S	S	S	S	S	S	S	S
HC10	R	S	S	S	S	S	S	S	S	S	S	S	S
IC11	S	S	S	S	S	S	S	S	S	S	S	S	S
HC11	R	R	R	S	S	S	S	R	S	S	R	S	S
IC12	R	R	R	R	R	R	R	R	S	S	S	S	S
HC12	R	R	R	R	S	R	R	R	S	S	S	S	S
IC13	R	R	R	R	S	R	S	S	S	S	S	S	S
HC13	R	R	R	R	S	S	S	S	S	S	S	S	S

Susceptibilities are reported in μg/mL.

Abbreviations: *INH* – isoniazid, *RIF* – rifampin, *EMB* – ethambutol, *PZA* – pyrazinamide, *STR* – streptomycin, *CAP* – capreomycin, *KAN* – kanamycin, *AMI* – amikacin, *ETH* – ethionamide, *CYC* – cycloserine, *PAS* – para-aminosalicylic acid; *R* – resistant, *S* – susceptible.

^*a*^With the exception of PZA, susceptibility for all drugs was performed by the 1% indirect agar proportion method. PZA susceptibility was performed on the VersaTREK (TREK Diagnostics, Cleveland, OH).

^*b*^Isolate unable to grow in test medium for PZA susceptibility testing.

Comparing test results of paired IC-HC isolates, only 3/16 (18.8%) had identical drug susceptibility results. In one household with four cases of TB (IC6), only two isolates had the same susceptibility pattern. Both isolates with the Beijing genotype were multidrug resistant, whereas 11 of 17 (64.7%) isolates with the Manila genotype were multidrug resistant.

## Discussion

DNA fingerprinting of *M tuberculosis* has transformed our understanding of the transmission of TB and provides a tool for the identification of transmission patterns. We sought to better understand TB transmission in the Philippines, which has a high TB and MDR TB burden, by molecular characterization of *M tuberculosis* isolates from household contacts of TB cases. Using three genotyping methods, our results show that based on the highest level of discrimination by IS6110 fingerprinting, approximately two-thirds (10/16, 62.5%) of TB in household contacts may have been acquired from a source outside of the household. A number of studies have elucidated that household contacts of TB patients have a higher risk of being infected [17-20]. In high TB prevalence setting, there are multiple sources of infection and numerous opportunities for TB transmission as a result of social mixing [21].

In Capetown, South Africa, where TB is highly endemic, RFLP analysis of 313 isolates from 129 households showed matching fingerprints in only 46%; the authors estimated that proportion of TB transmission occurring in household is only 19% [22]. Metro Manila is the largest city in the Philippines, with a population of over 11 million people, and a large number of urban poor settlements where TB is a substantial problem [23]. We analyzed *M tuberculosis* isolates that were collected from index and household contact TB cases in Metro Manila. IS6110 genotypes from household contacts matched those of index cases in six of 16 (37.5%). In the two households with more than two TB cases (IC6 and IC7), none of the isolates from the same household matched in IS6110 fingerprint. The fact that individuals with TB from the same household are infected with different strains of *M tuberculosis* suggests that the infection was acquired from outside the household, and most likely reflects the TB burden in the community.

Very few studies have been done to molecularly characterize *M tuberculosis* isolates from the Philippines. Analysis of 40 randomly collected isolates from the Philippines revealed that 38 of isolates had 80% or greater similarity, but non-identical, IS6110 fingerprints; these isolates has been given the designation as the Manila family [12]. In another study, 33 of 34 (97.0%) isolates from the Philippines also collected at random, showed similar but distinct IS6110 fingerprints [11]*. These* isolates had more than eight copies of IS6110, most having 13 copies. Our findings corroborate the presence of different strains of *M tuberculosis* with 11 or greater copies of IS6110 circulating in the community.

Spoligotyping of 48 *M tuberculosis* isolates from Manila showed patterns that were highly similar; 41/48 (85.4%) had identical patterns lacking hybridization to eight spacers [12]. These isolates have been classified as the Manila family. In our study on 29 isolates, 17 (58.6%) lacked hybridization to these spacers.

In The Gambia, spoligotyping on nine paired index-household cases revealed six (66.6%) pairs to match and three (33.3%) that were unmatched [24]. Our results showed a similar rate of spoligotype matching in 11 of 16 (68.8%) pairs. In the Philippines where most isolates of *M tuberculosis* have ≥8 copies of IS6110 [12], spoligotyping does not appear to be sufficiently discriminatory as an epidemiologic tool. We detected the same number of matched isolates (11/16) with MIRU-VNTR as spoligotyping.

IS6110 RFLP typing [25] has been replaced in some settings with easier-to-perform, alternative PCR-based strategies, such as spoligotyping and MIRU-VNTR. These alternative typing methods, with classical epidemiological investigations, are being internationally adopted as the new reference method for TB molecular epidemiology [26,27]. A recent population-based study indicated that the use of MIRU-VNTR system as a first-line method in combination with spoligotyping provides adequate discrimination in most cases for large-scale, prospective genotyping of *M. tuberculosis* in the United States [28]. However, IS*6110* fingerprinting is still useful as an additional method to type the clustered isolates in a number of cases. The combination of these two PCR-based typing methods may be useful in low TB prevalence settings [28], but its utility in high TB prevalence settings has yet to be clearly demonstrated. In our sample of 16 paired isolates for example, the combination of spoligotyping and MIRU-VNTR typing matched 10 pairs of isolates, whereas IS6110 fingerprinting further discriminated the isolates into only six matching pairs.

We tested a high number of MDR *M tuberculosis* isolates; this was expected since we identified patients at a referral center for treatment of MDR TB [29]. Comparing fingerprint data with drug susceptibility results, only three of the six (50.0%) pairs with identical genotypes had matching drug susceptibility. There were no observable patterns in terms of the matched isolates being from a particular type of household relationship (eg., the young child or the partner of an index case) or in the length of time between diagnosis of the index patient and the household contact(s) (Table 3). Since 10 of 13 index patients had MDR-TB, we were unable to determine whether MDR-TB patients were more likely to transmit TB to a household contact than the non-MDR index patients. These finding have implications in the treatment of TB and highlight the importance of drug susceptibility testing; treatment of TB in household contacts cannot be inferred from drug susceptibility of *M tuberculosis* of index patients.

**Table 3:** Patient demographics

**Index patient/ pousehold contact(s)**	**Year diagnosed with tuberculosis**	**Years between diagnosis**	**Age at diagnosis**	**Sex**	**Relationship**	**DST matches? (Y/N)**	**Genotyping match?**^**a **^**(Y/N)**
IC1	2001		41	F			
HC1	2001	0	22	F	child	N	Y
IC2	2001		28	M			
HC2	2001	0	18	M	sibling	N	N
IC3	2001		52	M			
HC3	NA	NA	22	F	child	N	Y
IC4	2001		38	M			
HC4	2001	0	10	M	child	N	N
IC5	2001		42	M			
HC5	2001	0	22	M	child	Y	Y
IC6	2002		17	M			
HC6.1	2002	0	20	M	sibling	N	N
HC6.2	2002	0	29	M	sibling	N	N
HC6.3	2002	0	22	M	sibling	N	N
IC7	2002		43	M			
HC7.1	2002	0	33	F	sibling	N	N
HC7.2	NA	NA	41	M	sibling	N	N
IC8	2001		28	F			
HC8	2007	6	60	M	parent	N	N
IC9	2001		19	M			
HC9	2003	2	30	M	sibling	Y	Y
IC10	2000		40	M			
HC10	2003	3	40	F	sibling	Y	Y
IC11	2003		NA	M			
HC11	2004	1	NA	M	NA	N	N
IC12	2003		30	F			
HC12	2004	1	28	F	sibling	N	N
IC13	2003		49	M			
HC13	2008	5	32	M	child	N	Y

*DST* = drug susceptibility testing, *NA* = not available, *Y* = yes, *N* = no.

^a^Using all 3 methods (spoligotyping, MIRU-VNTR and RLFP).

Our study has some limitations. We tested only a small number of isolates, and samples were collected from a TB referral center in one geographic area in the Philippines. Our results may not reflect the predominantly circulating strains of *M tuberculosis* in the general population. Lately, MIRU-VNTR typing of 15 or 24 loci has been applied to molecular typing of *M tuberculosis*; we may have found more genetic diversity in our samples with 24-loci method than with the 12-loci method used in this study. Nonetheless, our data demonstrate that there is significant genotype and drug susceptibility diversity within households with multiple cases of tuberculosis.

## Conclusions

Patients in high TB burden regions are exposed to TB outside the home, and therefore may be infected by source patients outside the home. Genotyping of *M tuberculosis* from the Philippines will aid in understanding the epidemiology of TB of this country, which can lead to improved strategies to reduce TB prevalence in the country.

Our results augment the findings from other studies that in highly prevalent areas, TB transmission is as likely to occur in the community as it is within the household. Furthermore, our data highlights the importance of drug susceptibility testing particularly for household contacts of MDR TB patients to ensure that they receive the appropriate drugs to which their isolates are susceptible. Our results have significant implications to TB control in the Philippines. Strain typing of TB provides valuable information on the number and type of strains circulating in a community; more importantly, it provides information on the transmission, which informs prevention and control. Further studies on a larger number of isolates are needed to properly elucidate the epidemiology and transmission of TB in this country.

## Competing interests

The authors declare that they have no competing interests.

## Authors’ contributions

IS conceived of the study, contributed to study design and coordination, and helped draft the manuscript. SB contributed to study design and coordination, performed DST and identifications, interpreted results and helped draft the manuscript. KD performed DST and interpreted results; SL performed genotyping and interpreted results, RK performed genotyping, interpreted results and helped draft the manuscript. R O-C performed identifications and DST testing; MQ performed identifications and DST testing; TT contributed to the study design, interpreted results and helped draft the manuscript. NW contributed to study design and coordination, interpreted results and helped draft the manuscript. All authors read and approved the final manuscript.

## Pre-publication history

The pre-publication history for this paper can be accessed here:

http://www.biomedcentral.com/1471-2334/13/571/prepub

## References

[B1] Corbett EL Watt CJ Walker N Maher D Williams BG Raviglione MC Dye C The growing burden of tuberculosis: global trends and interactions with the HIV epidemic Arch Intern Med 2003 13 9 1009 1021 10.1001/archinte.163.9.1009 12742798

[B2] Sia I Orillaza R St Sauver J Quelapio I Lahr B Alcaneses R Wilson W Cockerill F Balane G Mangubat N Tuberculosis attributed to household contacts in the Philippines Int J Tuberc Lung Dis 2010 13 1 122 125 20003706

[B3] WHO Global Tuberculosis Control Report 2012 Geneva, Switzerland: World Health Organization

[B4] Peabody JW Shimkhada R Tan C Jr Luck J The burden of disease, economic costs and clinical consequences of tuberculosis in the Philippines Health Policy Plan 2005 13 6 347 353 10.1093/heapol/czi041 16155066

[B5] Daley CL Molecular epidemiology: a tool for understanding control of tuberculosis transmission Clin Chest Med 2005 13 2 217 231 vi 10.1016/j.ccm.2005.02.005 15837107

[B6] Kamerbeek J Schouls L Kolk A van Agterveld M van Soolingen D Kuijper S Bunschoten A Molhuizen H Shaw R Goyal M Simultaneous detection and strain differentiation of Mycobacterium tuberculosis for diagnosis and epidemiology J Clin Microbiol 1997 13 4 907 914 9157152 10.1128/jcm.35.4.907-914.1997 PMC229700

[B7] van Soolingen D de Haas PE Hermans PW Groenen PM van Embden JD Comparison of various repetitive DNA elements as genetic markers for strain differentiation and epidemiology of Mycobacterium tuberculosis J Clin Microbiol 1993 13 8 1987 1995 7690367 10.1128/jcm.31.8.1987-1995.1993 PMC265684

[B8] Mazars E Lesjean S Banuls AL Gilbert M Vincent V Gicquel B Tibayrenc M Locht C Supply P High-resolution minisatellite-based typing as a portable approach to global analysis of Mycobacterium tuberculosis molecular epidemiology Proc Natl Acad Sci USA 2001 13 4 1901 1906 10.1073/pnas.98.4.1901 11172048 PMC29354

[B9] Frothingham R Meeker-O'Connell WA Genetic diversity in the Mycobacterium tuberculosis complex based on variable numbers of tandem DNA repeats Microbiology 1998 13 Pt 5 1189 1196 9611793 10.1099/00221287-144-5-1189

[B10] Allix C Supply P Fauville-Dufaux M Utility of fast mycobacterial interspersed repetitive unit-variable number tandem repeat genotyping in clinical mycobacteriological analysis Clin Infect Dis 2004 13 6 783 789 10.1086/423383 15472808

[B11] Park YK Bai GH Kim SJ Restriction fragment length polymorphism analysis of Mycobacterium tuberculosis isolated from countries in the western pacific region J Clin Microbiol 2000 13 1 191 197 10618086 10.1128/jcm.38.1.191-197.2000 PMC88694

[B12] Douglas JT Qian L Montoya JC Musser JM Van Embden JD Van Soolingen D Kremer K Characterization of the Manila family of Mycobacterium tuberculosis J Clin Microbiol 2003 13 6 2723 2726 10.1128/JCM.41.6.2723-2726.2003 12791915 PMC156522

[B13] Salazar-Vergara RM Sia IG Tupasi TE Alcaneses MR Orillaza RB Co V Quelapio MI Beltran G Legaspi JD Rostrata MP Tuberculosis infection and disease in children living in households of Filipino patients with tuberculosis: a preliminary report Int J Tuberc Lung Dis 2003 13 12 Suppl 3 S494 S500 14677843

[B14] Kent PT Kubica GP Public health mycobacteriology: a guide for the level III laboratory 1985 Centers for Disease Control and Prevention: Atlanta, GA

[B15] CLSI Susceptibility Testing of Mycobacteria, Nocardia and Other Aerobic Actinomycetes - Second Edition 2011 950 West Valley Road, Suite 2500 Wayne, PA 19087 USA: Clinical and Laboratory Standards Institute 31339680

[B16] de Beer J Kremer K Kodmon C Supply P Van Soolingen D Augustynowicz-Kopec E Bidovec Stojkovic U Boeree M Brown T Cirillo D First worldwide proficiency study on variable-number tandem-repeat typing of Mycobacterium tuberculosis complex strains J Clin Microbiol 2012 13 3 662 669 Epub 2011 Dec 2014 10.1128/JCM.00607-11 22170917 PMC3295139

[B17] Shaw J Wynn-Williams N Infectivity of pulmonary tuberculosis in relation to sputum status Am Rev Tuberc Respir Dis 1954 13 724 732 10.1164/art.1954.69.5.724 13148535

[B18] Lutong L Bei Z Association of prevalence of tuberculin reactions with closeness of contact among household contacts of new smear-positive pulmonary tuberculosis patients Int J Tuberc Lung Dis 2000 13 3 275 277 10751077

[B19] Fuimaono A Vince J Screening contacts of children with tuberculosis: an important and worthwhile part of case management P N G Med J 1997 13 2 69 73 10513226

[B20] Beyers N Gie RP Schaaf HS Van Zyl S Talent JM Nel ED Donald PR A prospective evaluation of children under the age of 5 years living in the same household as adults with recently diagnosed pulmonary tuberculosis Int J Tuberc Lung Dis 1997 13 1 38 43 9441057

[B21] Classen CN Warren R Richardson M Hauman JH Gie RP Ellis JH van Helden PD Beyers N Impact of social interactions in the community on the transmission of tuberculosis in a high incidence area Thorax 1999 13 2 136 140 10.1136/thx.54.2.136 10325918 PMC1745413

[B22] Verver S Warren RM Munch Z Richardson M van der Spuy GD Borgdorff MW Behr MA Beyers N van Helden PD Proportion of tuberculosis transmission that takes place in households in a high-incidence area Lancet 2004 13 9404 212 214 10.1016/S0140-6736(03)15332-9 14738796

[B23] Tupasi TE Radhakrishna S Quelapio MI Villa ML Pascual ML Rivera AB Sarmiento A Co VM Sarol JN Beltran G Tuberculosis in the urban poor settlements in the Philippines Int J Tuberc Lung Dis 2000 13 1 4 11 10654637

[B24] Hill PC Jackson-Sillah DJ Fox A Brookes RH de Jong BC Lugos MD Adetifa IM Donkor SA Aiken AM Howie SR Incidence of tuberculosis and the predictive value of ELISPOT and Mantoux tests in Gambian case contacts PLoS One 2008 13 1 e1379 10.1371/journal.pone.0001379 18167540 PMC2147055

[B25] van Embden JD Cave MD Crawford JT Dale JW Eisenach KD Gicquel B Hermans P Martin C McAdam R Shinnick TM Strain identification of *Mycobacterium tuberculosis* by DNA fingerprinting: recommendations for a standardized methodology J Clin Microbiol 1993 13 2 406 409 8381814 10.1128/jcm.31.2.406-409.1993 PMC262774

[B26] Supply P Allix C Lesjean S Cardoso-Oelemann M Rusch-Gerdes S Willery E Savine E de Haas P van Deutekom H Roring S Proposal for standardization of optimized mycobacterial interspersed repetitive unit-variable-number tandem repeat typing of Mycobacterium tuberculosis J Clin Microbiol 2006 13 12 4498 4510 10.1128/JCM.01392-06 17005759 PMC1698431

[B27] Oelemann MC Diel R Vatin V Haas W Rusch-Gerdes S Locht C Niemann S Supply P Assessment of an optimized mycobacterial interspersed repetitive- unit-variable-number tandem-repeat typing system combined with spoligotyping for population-based molecular epidemiology studies of tuberculosis J Clin Microbiol 2007 13 3 691 697 10.1128/JCM.01393-06 17192416 PMC1829086

[B28] Cowan LS Diem L Monson T Wand P Temporado D Oemig TV Crawford JT Evaluation of a two-step approach for large-scale, prospective genotyping of Mycobacterium tuberculosis isolates in the United States J Clin Microbiol 2005 13 2 688 695 10.1128/JCM.43.2.688-695.2005 15695665 PMC548083

[B29] Tupasi TE Quelapio MI Orillaza RB Alcantara C Mira NR Abeleda MR Belen VT Arnisto NM Rivera AB Grimaldo ER DOTS-Plus for multidrug-resistant tuberculosis in the Philippines: global assistance urgently needed Tuberculosis (Edinb) 2003 13 1–3 52 58 12758189 10.1016/s1472-9792(02)00072-0

